# Determination of nucleotide and enzyme degradation in haddock (*Melanogrammus aeglefinus*) and herring (*Clupea harengus*) after high pressure processing

**DOI:** 10.7717/peerj.7527

**Published:** 2019-08-27

**Authors:** Nurul Ulfah Karim, James Terence Kennedy, Mark Linton, Margaret Patterson, Sally Watson, Norman Gault

**Affiliations:** 1 School of Fisheries and Aquaculture Sciences, Universiti Malaysia Terengganu, Kuala Terengganu, Terengganu, Malaysia; 2 Agriculture, Food and Environmental Science Division, Agri-Food and Biosciences Institute, Belfast, UK

**Keywords:** Herring, Nucleotides degradation, Haddock, 5-nucleotidase, Nucleoside phosphorylase, Hypoxanthine

## Abstract

**Background:**

The degradation of nucleotides and their enzymes had been widely used to evaluate fish freshness. Immediately after fish death, adenosine triphosphate (ATP) degrades into inosine-5-monophosphate (IMP) via adenosine-5-diphosphate (ADP) and adenosine-5-monophosphate (AMP). IMP degradation continues to produce inosine (ino) and hypoxanthine (Hx) and further deteriorates the fish by producing xanthine and uric acid. The dephosphorylation of IMP to Ino is carried out by the enzyme 5′-nucleotidase (5′-NT), whereas the degradation of Ino to Hx is carried out by the enzyme nucleoside phosphorylase (NP). This study assesses the application of high pressure processing (HPP) in two species of fishes; haddock (*Melanogrammus aeglefinus*) and herring (*Clupea harengus*) as a means to extend the shelf-life by slowing down the rate of nucleotides degradation.

**Methods:**

Haddock *(Melanogrammus aeglefinus)* and herring *(Clupea harengus)* fillets were subjected to HPP at 200, 250 and 300 MPa for 1 and 3 min before being stored for 14 days. In addition, 5′-NT and NP enzyme activities were determined on both fish species that were subjected to 100–600 MPa for 1 and 3 min.

**Results:**

Adenosine triphosphate, ADP and AMP in both haddock and herring were lower at higher pressure levels. Inosine (Ino) increased (*p* < 0.05) after treatment at higher pressures in both species. Hx in herring decreased significantly (*p* < 0.05) at higher pressures but not in haddock. *K* values are the ratio of Ino and Hx to all nucleotides. *K* values in haddock were not significantly (*p* > 0.05) affected by the pressure treatment. *H* values are ratio of Hx to the sum of IMP, Ino and Hx. *H* values in haddock were significantly decreased (*p* < 0.05) with increasing pressure level. *F* values are ratio of IMP to the sum of IMP, Ino and Hx. *F* value*s* showed no significant effects (*p* > 0.05) after pressure treatment. Furthermore, *K* value*s* in control herring were significantly higher (*p* < 0.05) than those of the pressure-treated samples. *H* values in herring decreased significantly (*p* < 0.05) with increasing pressure level. *F* value*s* in herring showed no significant effects (*p* > 0.05) after pressure treatment. Pressure treatment brought a significant decrease (*p* < 0.05) in protein content in both haddock and herring. 5′-NT activity was 20–35 fold higher compared to NP activity in haddock and 15–44 fold higher than NP activity in herring. 5′-NT and NP activities decreased significantly with increasing pressure level in both species.

**Discussion:**

High pressure processing effectively slows down the conversion of Ino to Hx, delaying the undesirable flavour that develops in spoiling fish. The autolytic conversion of IMP to Ino by endogenous 5′-NT predominates in the earliest stages of storage is an autolytic process. However, both bacterial and endogenous NP enzymes are probably responsible for the gradual accumulation of Hx in fish. *K* value*s* are recommended as a useful measurement of fish freshness.

## Introduction

Nucleotides are a class of organic compounds comprising a nitrogen-containing base linked to a sugar and phosphate group (Whittle & Howgate, 2002). Adenosine triphosphate (ATP) is central to the metabolic processes of cells and essential for muscle contraction. In fish muscle, the pathway of ATP catabolism after death has been extensively documented as a sequential enzymatic degradation to adenosine-5-diphosphate (ADP), adenosine-5-monophosphate (AMP), inosine-5-monophosphate (IMP), inosine (Ino) and hypoxanthine (Hx). This degradation process begins immediately after death (Veciana-Nogues et al., 1996). The development of *rigor mortis* in fish muscle is a direct response to the decline of ATP (Borgstrom, 1961; Hong, Regenstein & Luo, 2017). Generally, ATP and ADP disappear around 24 h after death. AMP also decreases rapidly and its concentration becomes negligible. As a consequence, IMP and Ino increase sharply within 1–2 days after death (Lindsay, 1994; Lakshmanam & Gopakumar, 1999) but then decrease gradually as the concentration of Hx increases with further storage (Saito, Arai & Matsuyoshi, 1959; Park & Kim, 1999).

IMP and Hx are significant contributors to fish flavour, but in different ways. IMP is known to contribute to the pleasant flavour of fresh fish and is correlated with the desirable sweet and salty taste of fresh fish (Shahidi, Chong & Dunajski, 1994; Kawai, Okiyama & Ueda, 2002; Kuda et al., 2008; Hong, Regenstein & Luo, 2017). In contrast, Ino and Hx are associated with the progressive loss of the desirable flavour of fresh fish and unpleasant bitterness (Surette, Gill & Maclean, 1990; Haard, 2002; Li, Luo & Shen, 2015). The degradation of ATP to IMP is mainly attributed to endogenous autolytic enzyme activity (Gram & Huss, 1996), whereas the breakdown of IMP to Hx is attributed to both enzymatic and microbial activity. It is well documented that nucleotide degradation, particularly the loss of IMP and subsequent increase of Hx, which has been mathematically expressed as an increase in *K* values, the ratio of Ino and Hx to all nucleotides and their catabolic derivatives (Gill, 1995), is a good indicator of loss of freshness in fish (Ashie & Simpson, 1996; Howgate, 2005, 2006) and thus of storage age (Mendes, Quinta & Nunes, 2001).

Generally, *K* values of 20% represent an excellent degree of freshness for raw fish (sashimi grade) consumption (Tejada, 2009). *K* values of less than 30% have been associated with an excellent degree of freshness for barramudi (*Lates calcarifer*) (Williams et al., 1993), Nile perch (*L. niloticus*) (Williams et al., 1993) and milkfish (*Chanos chanos*) (Chiou, Yu & Shiau, 1995). While such low *K* values represent the highest levels of freshness, it would appear that the limit of acceptability is extremely variable between fish species. For example, hoki (*Macruronus novaezelandiae*) (Ryder et al., 1993), farmed gilthead seabream (*Sparus senegalensis*) (Huidobro, Pastor & Tejada, 2001; Tejada, Huidobro & Mohamed, 2006) and Senegalese sole (*Solea senegalensis*) (Tejada, De las Heras & Kent, 2006) all have a limit of sensory acceptability corresponding to *K* values of 30–45%. In contrast, other species have reported limits of acceptability corresponding to *K* values in excess of 60%. The limit of sensory acceptability has been found to correspond to *K* values of 60–70% in farmed seabass (*Dicentrarchus labrax*) (Tejada, Huidobro & Mohamed, 2006), farmed turbot (*Psetta maxima*) (Rodriguez et al., 2006) and wild turbot (*Scophthalmus maximus*) (Ozogul et al., 2006); 70–80% in Atlantic salmon (*Salmo salar*) (Erikson, Bayer & Sigholt, 1997); and to as high as 80% in European whitefish (*Coregonus wartmanni*) (Hattula, Kiesvaara & Moran, 1993).

*H* values are defined as the ratio of Hx to the sum of IMP, Ino and Hx and have been proposed as an indicator to monitor the accumulation of Hx, and thus bitter flavour development in fish (Jones, 1965). The limit of acceptability of *H* values in fish for human consumption has been suggested as 60% (Kuley, Ozogul & Ozogul, 2005; Ozogul et al., 2006). The dephosphorylasation of IMP to form Ino and Hx is carried out by 5′-nucleotidase (5′-NT) and nucleotidase phosphorylase, respectively. Fish are categorised as Ino-forming species when *H* values are much lower than *K* values. Hx-forming species, in contrast, characterise those species where *K* values and *H* values not are noticeably different (Luong et al., 1992). The ratio of IMP to the sum of IMP, Ino and Hx, defined as an *F* value, would be an appropriate quality indicator of nucleotide degradation in Ino-forming species. Alasalvar, Taylor & Shahidi (2002) recently showed that *F* values gave a better indication of cultured sea bream freshness than other nucleotide breakdown indices. Fish were considered to be unacceptable at an *F*-value of 10% and higher (Gill et al., 1987; Ozogul et al., 2006).

During storage, the dephosphorylation of IMP to Ino is carried out by the enzyme 5′-NT and this has been found to be highly correlated to a lowering of fish freshness and quality (Gill, 2000; Yamaguchi & Ninomiya, 2000; Li et al., 2016). 5′-NT is an alkaline phosphatase which has been found in soluble, membrane-bound and surface-located forms (Zimmermann, 1992; Marseno, Hori & Miyazawa, 1992). Theoretically, 5′-NT is involved in two principal and related pathways; the inactivation and catabolism of ATP and the formation of adenosine (Zimmermann, 1992). The extracellular inactivation of ATP represents a necessary step in its control as an intercellular mediator. The last step, catalysed by 5′-NT, in turn produces adenosine (Zimmermann, 1992). 5′-NT released adenosine is transported into the cells where it is used as a substrate by cytoplasmic enzymes.

Nucleoside phosphorylase (NP) catalyses the cleavage of the glycosidic bond of inosine to generate the purine base and ribose- or deoxyribose-1-phosphate (dR-1-P) (Kim, Cha & Parks, 1968; Bzowska, Kulikowska & Shugar, 2000). The phosphate anion attacks the C1 of the pentose and weakens the glycosidic bond. This causes a shift of electrons into the purine ring, resulting in a negatively charged purine intermediate and ribose-1-phosphate (R-1-P) or dR-1-P (Watanabe et al., 1984). NP catalyses the phosphorylytic cleavage of purine ribonucleosides, purine-2-deoxyribonucleosides and the analogue of purine nucleosides (Bzowska, Kulikowska & Shugar, 2000). NP might phosphorylase guanosine, inosine, 2-deoxyguanosine, 2-deoxyinosine and ribonucleosides of 8-azaguanine and 6-mercaptopurine (Kim, Cha & Parks, 1968). NP will probably not split adenosine or 2-deoxyadenosine unless the nucleosides are deaminated by adenosine deaminase.

High pressure processing (HPP) is a non-thermal processing technique that has been applied to fish in an attempt to extend shelf-life by inhibiting microorganisms and slowing down the rate of nucleotide degradation. The aim of this study was to investigate the effect of HPP on the extent of nucleotide degradation in haddock and herring during subsequent chilled storage in vacuum packs and estimating the shelf-life by limiting the *K* values to 80% and *H* values at 60% and predicting the initial freshness loss (*F* values) at 10%. Further investigation on the effect of HPP on the enzymatic changes involved in the nucleotide breakdown that leads to fish spoilage and on the hypothesis that nucleotide catabolism may be partly attributable to exogenous bacterial enzyme action were also carried out.

## Materials and Methods

### Sample preparation

Fish were harvested from the Irish Sea and transferred to the laboratory in ice within 6 h of landings. The fish fillets were treated in an Avure Quintus 35L capacity high pressure vessel (Avure Technologies, Västerås, Sweden). A total of 126 samples were pressure treated in batches at 200, 250 and 300 MPa for 1 and 3 min and stored in an isothermal refrigerated room (2 ± 0.5 °C) and immersed in ice at a 1:1 fish to ice ratio for 14 days. Meanwhile, for the enzyme experiment, samples were pressure treated in batches at 100, 200, 300, 400, 500 and 600 MPa, each for 1 and 3 min. The experiment was replicated on three different sampling trips.

### Nucleotides extraction

Nucleotides were extracted according to the method of Ryder (1985) with a minor modification. Nine ml of 1.2M perchloric acid solution was added to 5 ± 0.1 g fish muscle followed by 0.5 ml 10 mM uridine solution as an internal standard. The mixture was homogenised before centrifuged in a MSE Mistral 3000*i* at 2,200*g* for 10 min at 0 °C. A total of 10 ml aliquots were separated from the potassium perchlorate formed by decantation and filtration through a Millex 0.22 µm disposable syringe filter (mixed cellulose ester filter membrane).

### Nucleotide analysis, identification and quantification

Nucleotide analysis was performed using a Thermo-Separations HPLC consisting of a P2000 binary gradient pump, an AS1000 autosampler, a UV1000 detector as well as a SCH1000 degasser and an SN4000 throughout to a PC. Separation of nucleotides was obtained using a Thermo-electron five µm HyPurity Aquastar silica-based polar end-capped 150 × 4.6 mm column. A total of 50 mM KH_2_PO_4_ pH 2.5 was used as the mobile phase at a flow rate of one ml min^−1^. The identification of nucleotides was made by matching retention times with calibrated standard solutions. Standard curves for ATP, ADP, AMP, IMP, Ino and Hx were constructed in the zero to two mM range. All nucleotide standards were obtained from Sigma Chemical Company.

*K* values were calculated according to the following concentration ratio (Gill, 1995);

$$K\ {\rm{ values}}\,\left( \% \right){\rm{ }} = {\rm{ }}100{\rm{ }} \times {{\left( {\left[ {{\rm{Ino}}} \right]{\rm{ }} + {\rm{ }}\left[ {{\rm{Hx}}} \right]} \right)} \over {\left( {\left[ {{\rm{ATP}}} \right]{\rm{ }} + {\rm{ }}\left[ {{\rm{ADP}}} \right]{\rm{ }} + {\rm{ }}\left[ {{\rm{AMP}}} \right]{\rm{ }} + {\rm{ }}\left[ {{\rm{IMP}}} \right]{\rm{ }} + {\rm{ }}\left[ {{\rm{Ino}}} \right]{\rm{ }} + {\rm{ }}\left[ {{\rm{Hx}}} \right]} \right)}}$$

*H* values were calculated as follows (Luong et al., 1992);

$$H\ {\rm{ values}}\,\left( \% \right){\rm{ }} = {\rm{ }}100{\rm{ }} \times {{\left[ {{\rm{Hx}}} \right]} \over {\left( {\left[ {{\rm{IMP}}} \right]{\rm{ }} + {\rm{ }}\left[ {{\rm{Ino}}} \right]{\rm{ }} + {\rm{ }}\left[ {{\rm{Hx}}} \right]} \right)}}{\rm{ }}$$

*F* values were calculated as follows (Kuley, Ozogul & Ozogul, 2005);

$$F\ {\rm{ values}}\,\,\left( \% \right){\rm{ }} = {\rm{ }}100{\rm{ }} \times {\rm{ }}{{\left[ {{\rm{IMP}}} \right]} \over {\left( {\left[ {{\rm{IMP}}} \right]{\rm{ }} + {\rm{ }}\left[ {{\rm{Ino}}} \right]{\rm{ }} + {\rm{ }}\left[ {{\rm{Hx}}} \right]} \right)}}{\rm{ }}$$

### Estimation of shelf-life

The estimated shelf lives using *K* values were analysed as for a randomised block experiment with seven treatments and the three replicates as blocks. The shelf-life was taken as the time for the *K* values to reach 80% (the limit of acceptability for consumer consumption) (Hattula, Kiesvaara & Moran, 1993). The shelf-life were estimated by fitting the data to an exponential curve constrained to have an upper asymptote of 100%. The same principle was used to estimate the loss of quality with regard to *F* values and *H* values at 10% and 60% upper acceptability limits respectively.

### Enzyme extractions

Extracts of crude enzymes were prepared from the fish samples by the method described by Marseno, Hori & Miyazawa (1992) with a minor modification. A total of 30 ml of 40 mM Tris buffer containing 20 mM magnesium chloride and 25 mM sodium chloride at pH 7.4 were added to 10 ± 0.1 g of fish muscle. The mixture was homogenised before centrifuged at 2,500×*g* for 30 min at 4 °C (MSE Mistral 3000i centrifuge). The supernatant was filtered through glass wool into a 50 ml cylinder and the volume recorded.

### Preparation of standards and assay reagents

Protein concentrations of the crude enzyme extracts were estimated by reference to a standard absorbance curve obtained from a set of diluted albumin standard (BSA) (2.0 mg ml^−1^ in a 0.9% aqueous NaCl solution containing sodium azide; Pierce, Rockford, IL, USA). The standard curve was prepared by plotting the absorbance for each BSA standard at 595 nm, corrected for the average of the blank readings, against its concentration in μg ml^−1^.

### 5′-nucleotidase enzyme assay

5′-nucleotidase (EC 3.1.3.5) activity was measured according to the method of Marseno, Hori & Miyazawa (1992) with a minor modification. Each fish protein homogenate was diluted to one mg ml^−1^ protein with 40 mM Tris-Hydrochloride buffer pH 7.5 containing one mM magnesium chloride and five mM dithiothreitol. A total of 50 μl aliquot of the diluted extract was then added to 400 μl of 40 mM Tris hydrochloride buffer pH 7.5 containing 20 mM magnesium chloride, 110 mM sodium chloride and 20 mM sodium β-glycerolphosphate. The mixtures were incubated at 40 °C for 10 min. The assay reaction was started by the addition of 50 μl of 30 mM IMP and the mixture then incubated at 40 °C for 30 min. The reaction was stopped by adding 500 μl of a mixture of 2.5% ammonium molybdate, 10% ascorbic acid and 2.5M hydrochloric acid. The concentration of active enzyme was determined based on its activity in units obtained from the inorganic phosphate standard graph. One unit of enzyme activity was defined as that corresponding to the release of one μmole of Pimin^−1^, and specific activity was defined as units per mg of protein.

### Nucleoside phosphorylase enzyme assay

The enzyme assay for NP (EC 2.4.2.1) was prepared according to the Sigma–Aldrich method (Sigma-Aldrich, 1994). Each fish protein homogenate was diluted to one mg ml^−1^ protein with purified water. The solutions were thoroughly mixed and measured for 10 min in a SP6-500 UV Spectrophotometer (Pye Unicam Ltd, Cambridge, England). The ∆A_293 nm_/min was calculated using the maximum linear rate for both the samples and the blanks.

The units of enzyme activity mg^−1^ crude protein were calculated as follows;

Units of enzyme activity/mg protein = (∆A_293 nm_/min Samples − ∆A_293 nm_/min Blank)/(12.0) (mg crude protein/ml reaction mix)

where 12.0 = the millimolar extinction coefficient of uric acid at 293 nm

The unit definition of enzyme activity was as follows;

One unit of NP will cause the phosphorolysis of 1.0 μmole of inosine to hypoxanthine and R-1-P per minute at pH 7.4 at 25 °C.

### Statistical analysis

The entire experiment was replicated three times for each species of fish. Data were analysed statistically using GenStat 12.1 (VSN International Ltd, Hemel Hempstead, Hertforshire, UK). In all analyses by two way ANOVA the residuals were inspected for normality and homogeneity of variance across the treatments. This showed that percentages of *K* values, *H* values and *F* values were normally distributed. The data for the thirteen treatments were analysed as for a randomised block experiment, comprising a control plus a factorial arrangement of six pressure levels (100–600 MPa) and two hold time (1 and 3 min).

## Results

### Nucleotide degradation in haddock

**Figure 1:**
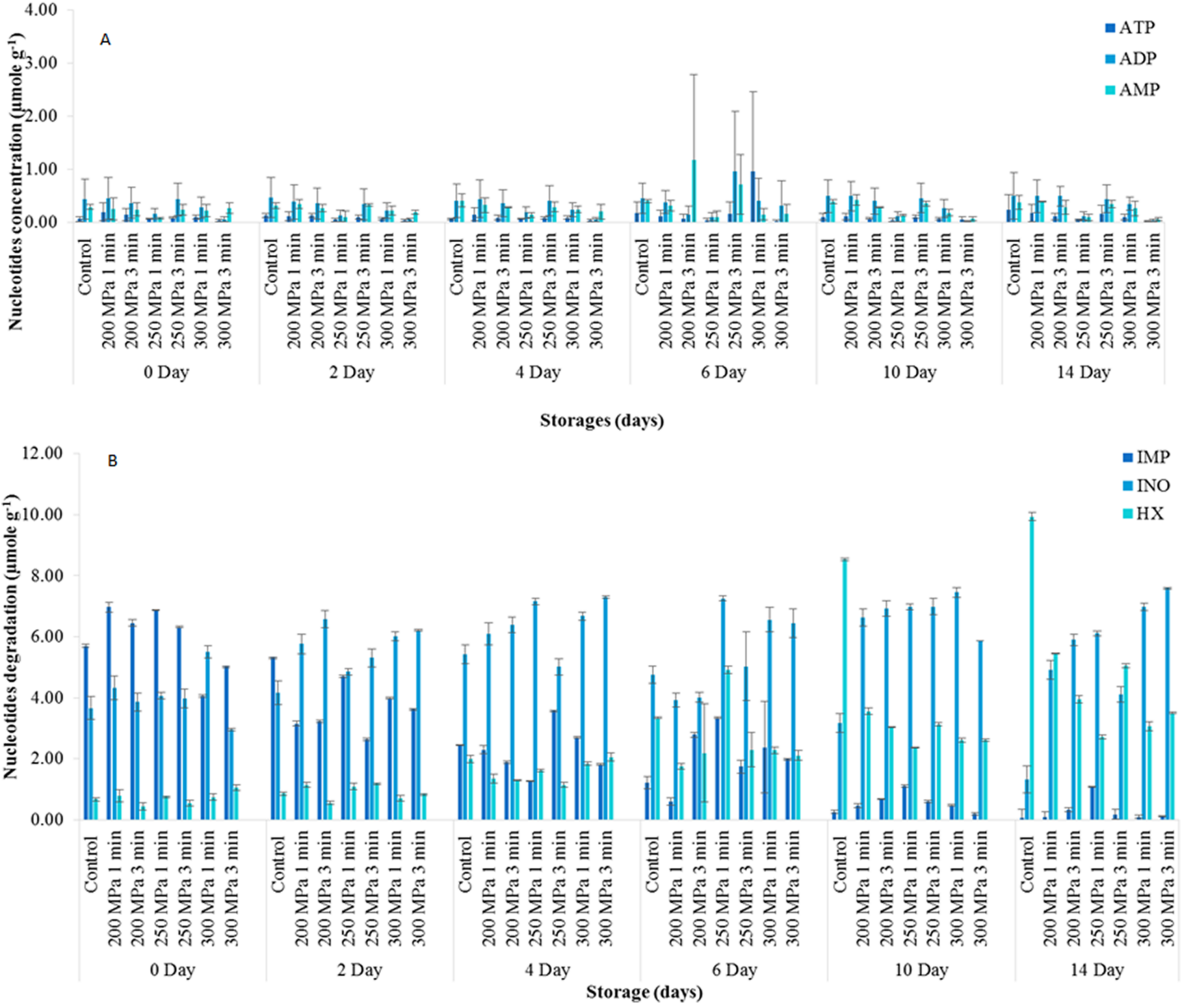
Nucleotides degradations (µmole g^−1^) of haddock untreated and treated in high pressure processing during 14 days of storage. (A) shows the degradation of ATP, ADP and AMP of haddock, and (B) shows the degradation of IMP, Inosine and Hypoxanthine of haddock during 14 days of storage. Data expressed as mean values ± standard deviation, *n* = 3. ADP, adenosine diphosphate; ATP, adenosine triphosphate; AMP, adenosine.

The changes of ATP, ADP and AMP in controls were negligible throughout the 14 day storage period (Fig. 1A). In contrast, IMP and Ino had relatively high concentrations on the first day of sampling. IMP decreased gradually after day 2 and was negligible by day 10. Ino increased from 3.66 ± 0.54 µmole g^−1^ on day 1 to 5.42 ± 0.90 µmole g^−1^ by day 4 and then fell to 1.32 ± 1.60 µmole g^−1^ by day 14. Hx increased steadily from a low of 0.67 ± 0.17 µmole g^−1^ on day 0 to a high of 9.93 ± 5.49 µmole g^−1^ by the end of the 14 days storage (Fig. 1B).

Neither pressure level, hold time nor storage time had significant effects (*p* > 0.05) on mean ATP concentrations. ADP and AMP concentrations were significantly lower (*p* < 0.05) in haddock pressure treated at 300 MPa compared haddock treated at 200 and 250 MPa (Fig. 1A). In addition, neither pressure level nor hold time had any significant effect (*p* > 0.05) on IMP concentrations. Haddock treated at 200 MPa had a significantly lower (*p* < 0.05) concentration of Ino compared to pressure treatments at 250 and 300 MPa (Fig. 1B). However, neither hold time nor storage time had a significant effect (*p* > 0.05) on mean Ino concentrations. In addition, Hx concentrations were not significantly affected (*p* > 0.05) by pressure level or pressure hold time (Fig. 1B).

There were no significant interactions (*p* < 0.05) between the controls and treated samples for ATP, ADP, AMP and IMP during 14 days of iced storage. However, a significant interaction (*p* < 0.05) was found between the controls and pressure-treated samples for Ino concentration. This was primarily due to the significantly higher (*p* < 0.05) Ino in the pressure treated samples compared to the controls from day 10 of storage. A similar but converse interaction was found with regard to Hx concentration. The concentrations of Hx were significantly higher (*p* < 0.05) in the controls compared to the pressure-treated samples from day 10 of chilled storage (Fig. 1B).

### *K* values, *H* values and *F* values in haddock after high pressure processing

**Figure 2:**
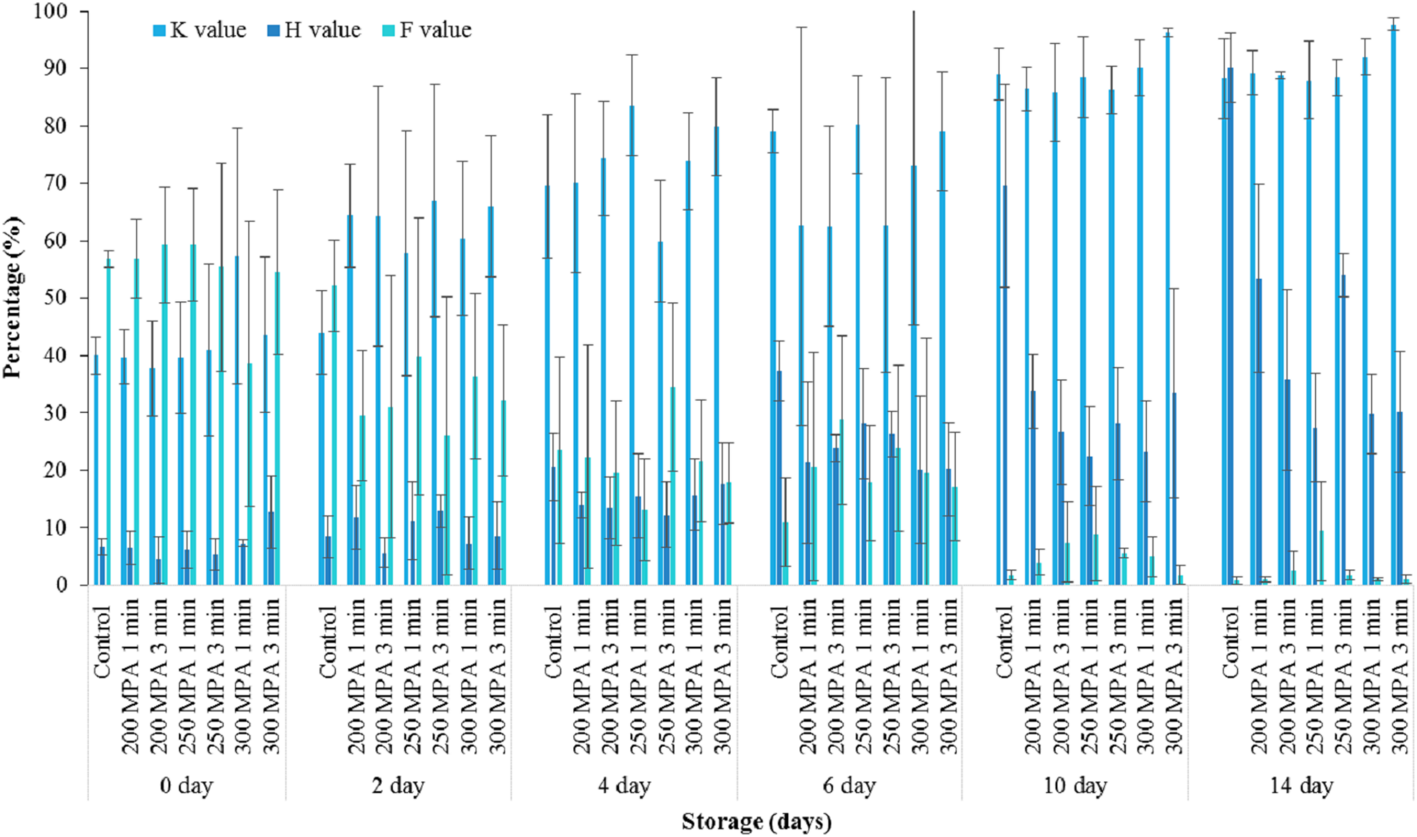
*K* values, *H* values and *F* values of haddock during 14 days of storage. Data expressed as mean values ± standard deviation. Mean *K*-, *H*-, and *F*-values calculated from the individual nucleotide datasets for the respective replicates.

*K* values in control haddock were not significantly different (*p* > 0.05) to those of the pressure-treated samples. Likewise, neither pressure level nor hold time had any significant effect (*p* > 0.05) effect on *K* values (Fig. 2).

Regardless of pressure level and hold time, the accumulation of Hx during 14 days of chilled storage was lower compared to the Ino. *H* values in haddock are influenced by the Ino concentration and thus, haddock can be categorised as Ino forming species. *H* values in controls were significantly higher (*p* < 0.05) than in pressure-treated samples by a factor of approximately 2. *H* values also decreased significantly (*p* < 0.05) with increasing pressure, from 23.29 ± 4.69% at 200 MPa to 19.47 ± 3.66% at 300 MPa. However, there was no significant difference (*p* > 0.05) in the mean *H* values of haddock treated at 250 and 300 MPa (Fig. 2).

*F* values in controls were not significantly different (*p* > 0.05) to those of the pressure-treated samples. Likewise, neither pressure treatment nor pressure hold time had any significant effect (*p* > 0.05) on *F* values in haddock (Fig. 2).

### Estimating the shelf-life of haddock from nucleotide ratios

Predicted *K* values in controls were 37.02% on day 0 and increased to the unacceptable standard of 80% around day 6 (Table 1). The comparison of estimated shelf lives based on *K* values for the controls and pressure treatments are between 4 and 7 days, although the differences between these treatments were not significant (*p* > 0.05). The same approach to estimating the shelf-life of haddock was determined using the *H* values. The controls reached the limit of acceptability for *H* values of 60% by day 9 (Table 1). Interestingly, pressure treatment of haddock at 300 MPa for 1 or 3 min extended the shelf-life, based on *H* values, to 74 days. Estimations of haddock shelf life based on *F* values indicated that haddock were considered to have reached the unacceptable *F* value freshness standard of 10% at between 5 and 8 days of storage. The initial deterioration of haddock was predicted to have started at day 5 in the controls, similar (*p* > 0.05) to that of many of the pressure treated samples (Table 1).

**Table 1:** A comparison of the estimated shelf-life of haddock fillets after various pressure treatments based on exponential fits of datasets for *K* values, *H* values and *F* values.

Treatment	Estimated shelf-life using *K* values (days to *K* = 80%)	Estimated shelf-life using *H* values (days to *H* = 60%)	Estimated shelf-life using *F* values (days to *F* = 10%)
Control	5.84	9.40	5.36
200 MPa 1 min	6.77	17.40	5.33
200 MPa 3 min	7.09	46.70	5.38
250 MPa 1 min	6.54	26.20	5.80
250 MPa 3 min	5.43	57.50	6.79
300 MPa 1 min	4.96	74.00	4.82
300 MPa 3 min	4.33	74.40	7.80
LSD	3.28	35.94	5.59

### Nucleotide degradation in herring

**Figure 3:**
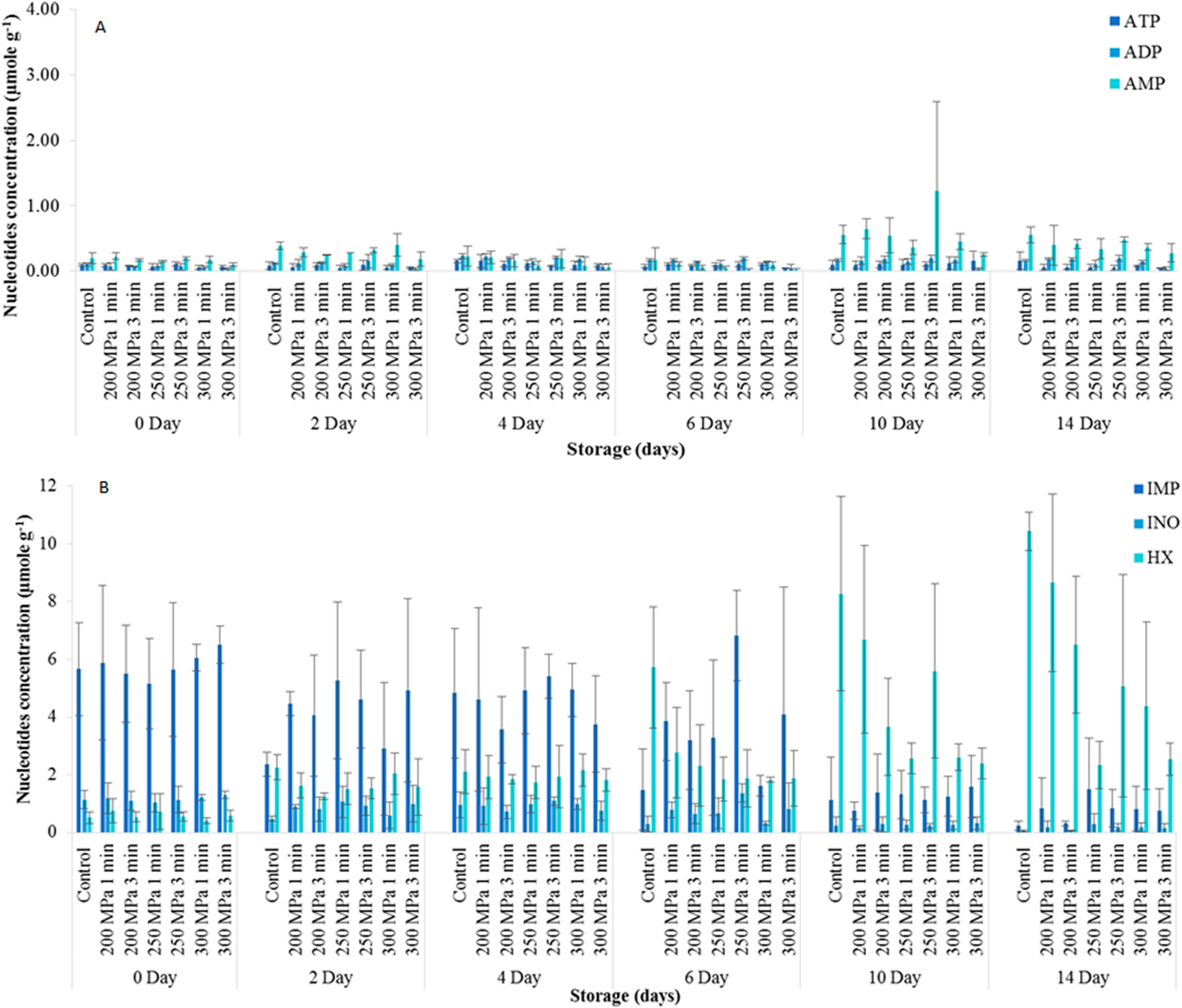
Nucleotide degradations (µmole g^−1^) of herring untreated and treated in high pressure processing during 14 days of storage. (A) shows the degradation of ATP, ADP and AMP of herring, and (B) shows the degradation of IMP, Inosine and Hypoxanthine of herring during 14 days of storage. Data expressed as mean values ± standard deviation, *n* = 3. ADP, adenosine diphosphate; ATP, adenosine triphosphate; AMP, adenosine.

The changes of ATP, ADP and AMP in controls were negligible throughout the 14 days storage period (Fig. 3A). In contrast, IMP decreased gradually from a high of 7.54 ± 1.62 μmole g^−1^ at the start of the storage period to a low of 0.23 ± 0.17 μmole g^−1^ after 14 days of storage (Fig. 3B). As nucleotide degradation progressed during storage, Ino concentration rose from 5.51 ± 1.00 μmole g^−1^ on day 0 to peak at 7.49 ± 0.37 μmole g^−1^ on day 4 before gradually decreasing to a low of 1.08 ± 0.99 μmole g^−1^ after 14 days of storage. In contrast, Hx increased steadily from 0.68 ± 0.21 μmole g^−1^ on day 0 up to 10.43 ± 0.67 μmole g^−1^ during 14 days of storage (Fig. 3B).

Neither pressure level, hold time nor storage time had any significant effect (*p* > 0.05) on ATP concentrations. ADP concentrations were also significantly decreased (*p* < 0.05) with increasing pressure level, although this was only apparent for the 300 MPa samples. However, AMP concentration in herring decreased significantly (*p* < 0.05) with increasing pressure level. Pressure hold time had no significant effect (*p* > 0.05) on AMP concentrations (Fig. 3A). In addition, neither pressure level nor pressure hold time had significant effect (*p* > 0.05) on IMP concentrations (Fig. 3B).

Ino concentrations increased significantly (*p* < 0.05) with increasing pressure level. Five Pressure hold time had no significant effect (*p* > 0.05) on the Ino concentration of herring. Hx concentration decreased significantly (*p* < 0.05) with increasing pressure treatment, from a high of 3.28 ± 1.58 µmole g^−1^ in the 200 MPa to a low of 1.82 ± 0.62 µmole g^−1^ in the 300 MPa. In addition, pressure hold time had no significant effect (*p* > 0.05) on Hx concentration in herring.

There were no significant interactions (*p* < 0.05) between the controls and treated samples of herring for ATP, ADP, AMP and IMP during 14 days of iced storage. However, significant interactions (*p* < 0.05) were found between the controls and pressure-treated samples for Ino and Hx concentrations. For Ino, this was due to the significantly higher (*p* < 0.05) mean concentration of Ino in the pressure treated samples compared to the controls from day 10 of storage. A similar but converse interaction was found with regard to Hx concentration. The mean concentrations of Hx were significantly higher (*p* < 0.05) in the controls compared to the pressure-treated samples from day 6 of chilled storage (Fig. 3B).

### *K* values, *H* values and *F* values in herring after high pressure processing

**Figure 4:**
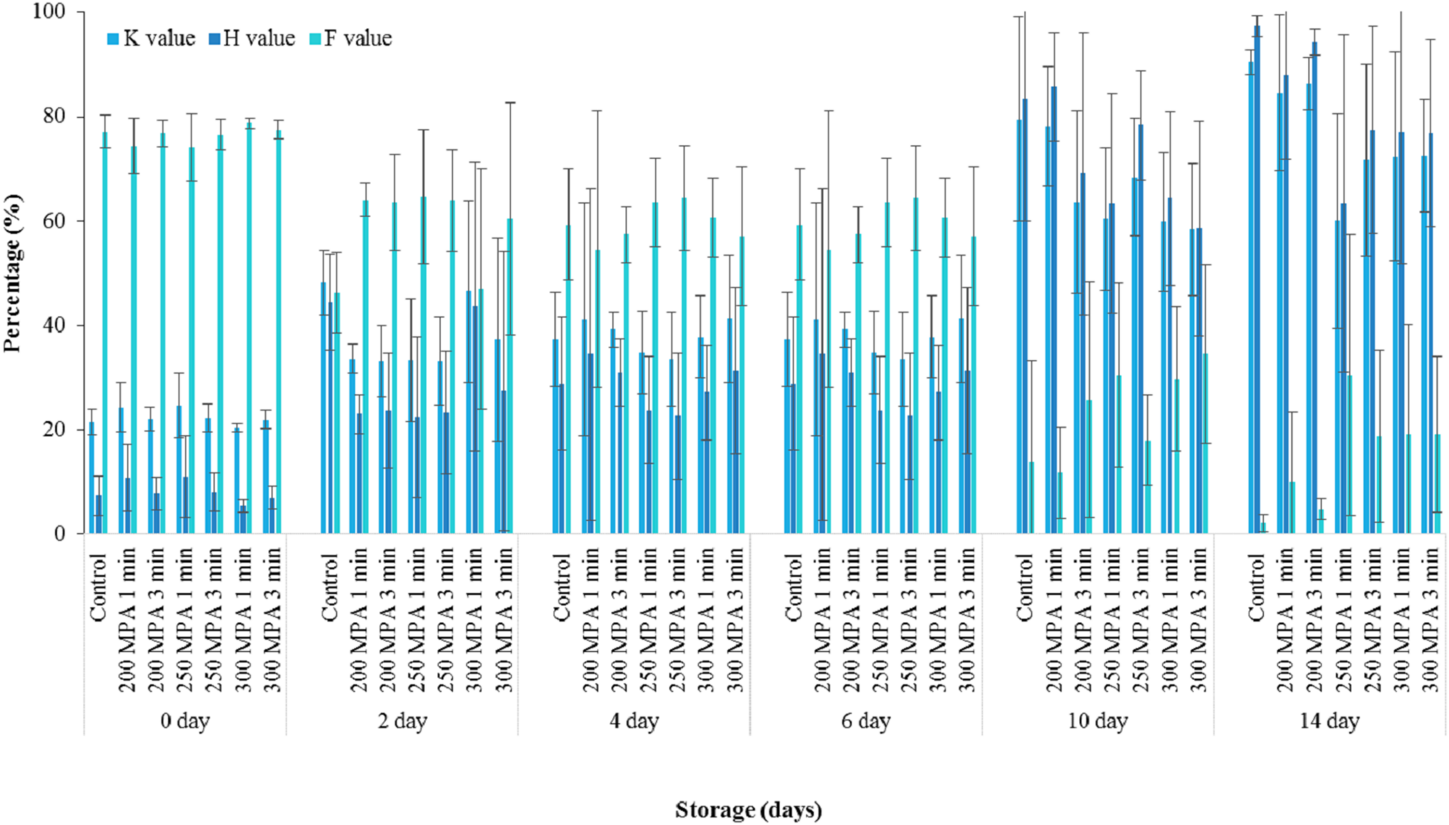
*K* values, *H* values *and F* values of herring during 14 days of storage. Data expressed as mean values ± standard deviation. Mean *K*-, *H*-, and *F*-values calculated from the individual nucleotide datasets for the respective replicates.

*K* values in control herring were significantly higher (*p* < 0.05) than those of the pressure-treated samples. However, neither pressure level nor hold time had any significant effect (*p* > 0.05) on *K* values in herring (Fig. 4).

Initially, herring contained 5.51 ± 1.00 µmole g^−1^ of Ino and 0.68 ± 0.21 µmole g^−1^ of Hx to give a ratio of Ino:Hx greater than 5:1. Ehira & Uchiyama (1973) stated that when Ino accumulation in fish is more than 5:1 times that of Hx concentration, the fish are considered as Ino-forming species. Therefore, herring can be categorised as an Ino-forming species. *H* values in controls were significantly higher (*p* < 0.05) than in pressure-treated samples by a factor of approximately 2. *H* values decreased significantly (*p* < 0.05) with increasing pressure level. Pressure hold time, however, had no significant effect (*p* > 0.05) on *H* values. *F* values in control herring were not significantly different (*p* > 0.05) to the pressure treated samples. Likewise, neither pressure level nor pressure hold time had any significant effect (*p* > 0.05) on *F* values in herring (Fig. 4).

### Estimating the shelf-life of herring from nucleotide ratios

Predicted *K* values in the controls were approximately 49.44% at the start of storage and increased to the 80% limit of acceptability by day 5. Meanwhile, predicted *K* values in the pressure-treated herring were around 46.95% at the start of storage and increased to the limit of acceptability by day 7. The estimated shelf lives for herring based on *K* values for the controls and the range of pressure treatments tested shows estimated shelf lives of between 5 and 10 days, although the differences between these treatments were spurious and not significant (*p* > 0.05) (Table 2).

**Table 2:** A comparison of the estimated shelf-life of herring fillets after various pressure treatments based on exponential fits of datasets for *K* values, *H* values and *F* values.

Treatments	Estimated shelf-life using *K* values (days to *K* = 80%)	Estimated shelf-life using *H* values (days to *H* = 60%)	Estimated shelf-life using *F* values (days to *F* = 10%)
Control	5.09	9.10	6.62
200 MPa 1 min	6.99	21.7	6.86
200 MPa 3 min	10.65	21.2	9.87
250 MPa 1 min	6.40	34.9	9.10
250 MPa 3 min	5.23	44.5	6.71
300 MPa 1 min	8.05	49.1	9.11
300 MPa 3 min	6.71	30.4	8.36
LSD	4.47	18.03	5.96

The same approach in estimating the shelf-life of herring was determined using the *H* values. The controls reached the limit of acceptability for *H* values of 60% by day 9. In contrast, pressure treatment of herring at 300 MPa for 1 min extended the estimated shelf-life up to 49 days. Estimations of herring shelf life based on *F* values were considered to have reached the unacceptable *F* value freshness standard of 10% at between 6 and 9 days of storage, although differences between controls and pressure treated herring were spurious and not significant (*p* > 0.05) (Table 2).

### Protein content of haddock enzyme extracts

**Figure 5:**
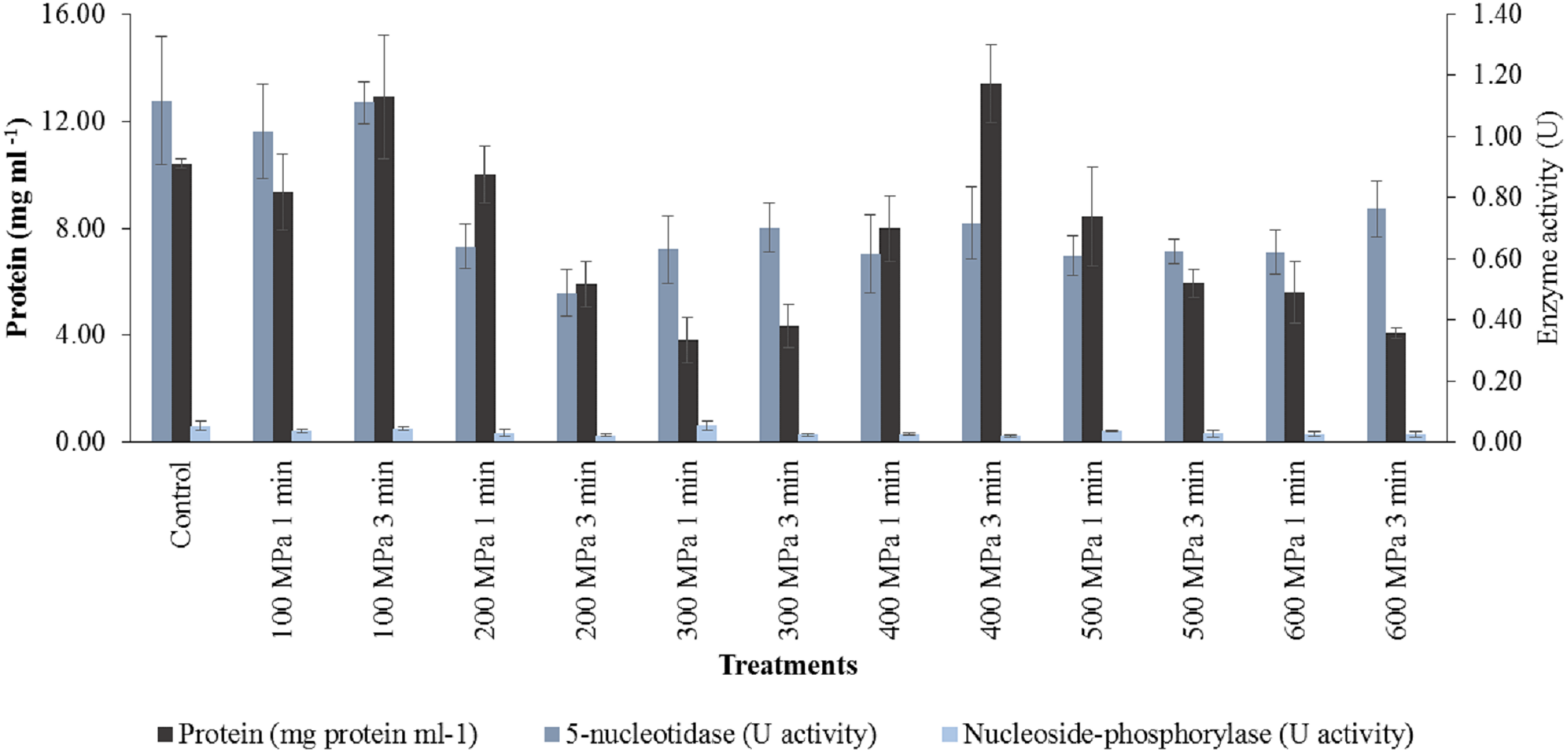
Protein content and enzyme activity in haddock at treatment of 100–600 MPa. Data expressed as mean values ± standard deviation.

Protein content of the untreated haddock extracts was 10.40 ± 0.17 mg protein ml^−1^, which were significantly higher (*p* < 0.05) than that of overall mean of the pressure-treated samples. Pressure treatment brought about a significant decreased (*p* < 0.05) in protein content in haddock extracts, from 8.88 ± 1.07 mg protein ml^−1^ at 100 MPa to 4.22 ± 0.54 mg protein ml^−1^ at 600 MPa. However, the highest protein content of 12.20 ± 0.74 mg protein ml^−1^ was found in the haddock treated at 200 MPa. The protein content of the haddock crude enzyme extracts were not significantly affected (*p* > 0.05) by pressure hold time (Fig. 5).

### 5′-nucleotidase activity in haddock extracts

High pressure processing resulted in a significantly lower (*p* < 0.05) mean 5′-NT activity in the pressure treated samples compared to the controls. 5′-NT activity significantly (*p* < 0.05) decreased with increasing pressure level, from 1.08 ± 0.09 U at 100 MPa to 0.69 ± 0.10 U at 600 MPa. However, there were no significant differences in 5′-NT activity at pressures from 200 to 600 MPa. The activity of 5′-NT was not significantly affected (*p* > 0.05) by pressure hold time (Fig. 5).

### Nucleoside-phosphorylase activity in haddock extracts

High pressure processing brought about a significant lowering (*p* < 0.05) of mean NP activity in haddock compared to controls. Pressure level brought about a significant decreased (*p* < 0.05) in NP activity, from 0.04 ± 0.01 U at 100 MPa to 0.02 ± 0.00 U at 600 MPa. However, there were random changes in NP activity at pressures from 200 to 600 MPa. An increase in pressure hold time from 1 to 3 min also brought about a significant decreased (*p* < 0.05) in mean NP activity (Fig. 5).

### Protein content of herring enzyme extracts

**Figure 6:**
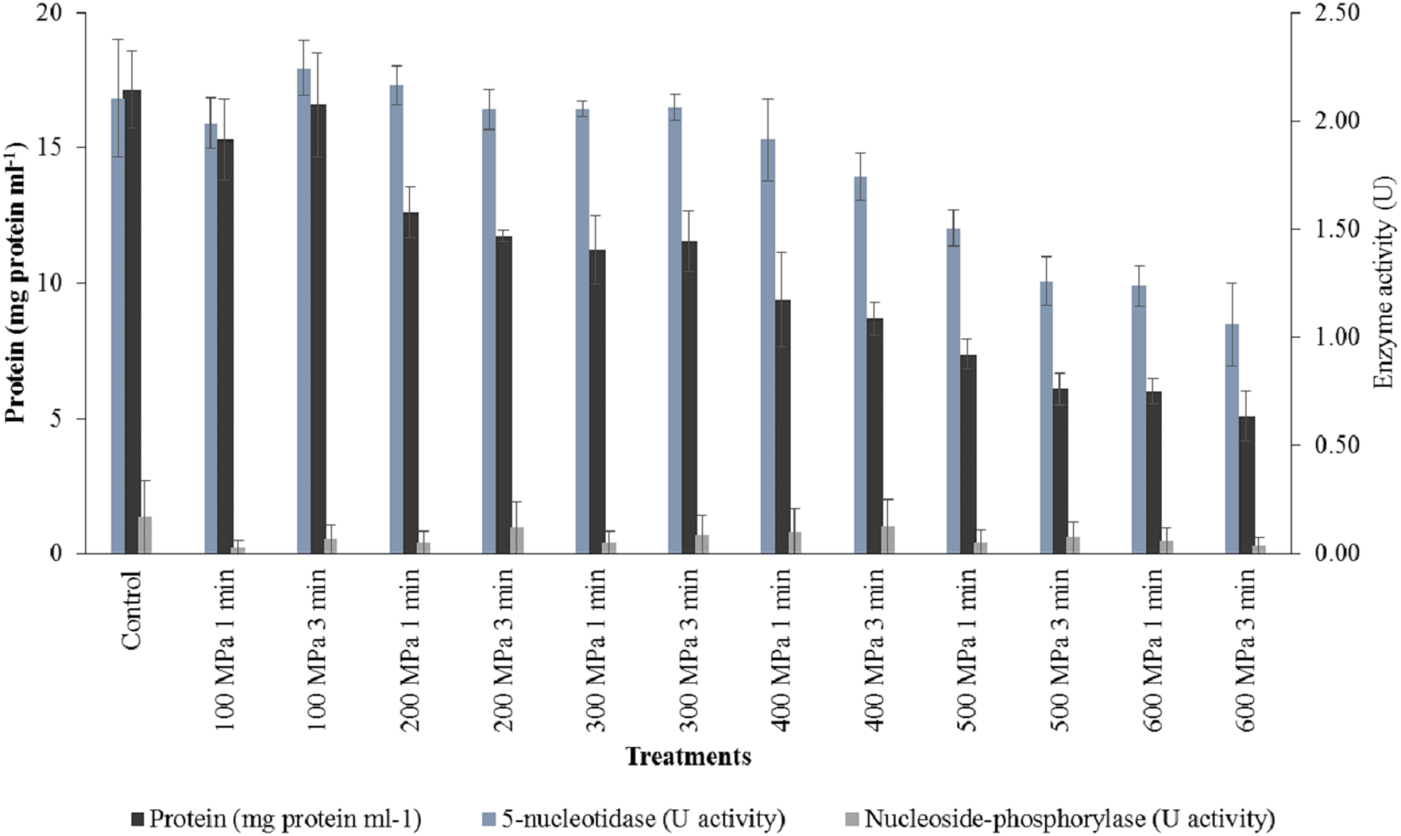
Protein content and enzyme activity in herring at treatment of 100–600 MPa. Data expressed as mean values ± standard deviation.

The protein content of the crude enzyme extract of the controls of herring was significantly higher (*p* < 0.05) than that of the pressure-treated samples. Protein content decreased significantly (*p* < 0.05) as pressure level increased, falling from 15.95 ± 0.89 mg protein ml^−1^ at 100 MPa to 5.54 ± 0.64 mg protein ml^−1^ after treatment at 600 MPa, although there was no significant decreased in concentration until 400 MPa. Pressure hold time had no significant effect (*p* > 0.05) on the protein content of the crude enzyme extracts of herring (Fig. 6).

### 5′-nucleotidase activity in herring extracts

5′-nucleotidase activity of the crude enzyme extracts of the control herring were significantly higher (*p* < 0.05) than that of the pressure-treated herring. 5′-NT activity decreased significantly (*p* < 0.05) with increasing pressure level beyond 300 MPa falling from 1.83 ± 0.15 U at 400 MPa to 1.11 ± 0.18 U at 600 MPa. At pressures between 100 and 300 MPa, pressure hold time had no significant effect (*p* > 0.05) on 5′-NT activity. However, 5′-NT activity was significantly higher in herring treated for 1 min than for 3 min at pressures between 400 and 600 MPa (Fig. 6).

### Nucleoside phosphorylase activity in herring extracts

Nucleoside phosphorylase activity of the crude enzyme extracts of the controls were significantly higher (*p* < 0.05) than that of the pressure-treated herring. NP activity changed significantly (*p* < 0.05) with increasing pressure level in a peculiar manner. Between 100 and 400 MPa, NP activity increased from 0.05 ± 0.03 U to peak at 0.11 ± 0.02 U, and decreased thereafter to a mean value of 0.05 ± 0.01 U at 600 MPa. NP activity was also significantly higher (*p* < 0.05) after the prolonged pressure hold time of 3 min (Fig. 6).

## Discussion

Lower concentrations of ATP, ADP and AMP were found in both haddock and herring after being processed at higher pressures, but no significant effect (*p* > 0.05) of pressure hold time on these nucleotide concentrations was found. In both species, during subsequent chilled storage, ATP, ADP and AMP gradually decreased further and became negligible by the end of the storage period. Watabe, Kamal & Hashimoto (1991) reported that ATP levels in sardine (*Sardinops melanostica*) almost disappeared after 12 h of iced storage. Mohan et al. (2009) found that ADP and AMP levels in seer fish (*Scomberomorus commerson*) were very low at the beginning of storage and decreased further over a 30 day storage period. Low levels of ATP, ADP and AMP were also found in Atlantic herring stored on ice in modified atmosphere and vacuum packs (Ozogul, Polat & Ozogul, 2004).

IMP has been identified as a flavour enhancer and is strongly associated with the acceptability of fish (Bremner et al., 1988; Mohan et al., 2009). Pressure level and hold time in haddock and herring had no significant (*p* > 0.05) effect on IMP concentration. However, the concentration of IMP in all treatments for both haddock and herring decreased significantly (*p* < 0.05) throughout the 14 days of chilled storage. IMP concentrations were marginally higher in herring than in haddock. Similar results were also found by Grigorakis, Taylor & Alexis (2003) in cultured sea bream stored on ice. Kuley, Ozogul & Ozogul (2005) found that IMP in sea bream stored in ice and wrapped with cling film and aluminium foil decreased throughout 15 days of storage. Alasalvar et al. (2001) also reported IMP value of cultured sea bream steadily decreased during 23 days of ice storage.

Interestingly, Ino concentrations were significantly higher (*p* < 0.05) after treatment at higher pressures in both haddock and herring, although pressure hold time had no effect (*p* > 0.05). Ino concentration in all treatments for both species was relatively as the same level during 14 days of chilled storage. In contrast, Hx concentrations in herring were significantly lower (*p* < 0.05) after increasing pressure treatment, but no significant trends (*p* > 0.05) were found in haddock. These trends nevertheless would suggest that high pressure treatment slows down the conversion of Ino to Hx compared to the untreated samples. Since increased concentrations of Hx are responsible for the undesirable flavour that develops in spoiling fish, the application of high pressure should extend the shelf-life of the sensory characteristics of these fish.

Neither pressure level nor pressure hold time had any significant effect (*p* > 0.05) on the *K* values of haddock or herring. However, during 14 days of chilled storage, *K* values increased significantly (*p* < 0.05) in both species. The rapid increase in *K* values is due to the breakdown of IMP during storage and the accumulation of steady levels of Ino and its slow conversion to Hx in both haddock and herring. Based on the *K* values for both haddock and herring, the shelf-life of haddock treated at 200 MPa for 3 min had the longest estimated shelf-life of up to 7 days, 2 days longer than the control. With herring, treatment at 200 MPa for 3 min and 300 MPa for 1 min gave extended shelf-lives for herring of 10 and 8 days respectively. However, considering the adverse effects of higher pressure on the appearance of the fish, 200 MPa for 3 min would be the best pressure treatment to apply to haddock and herring with regard to prolonging shelf-life based on *K* values.

*H* values were influenced by pressure level but not pressure hold time in both species. Storage period also produced significant increases (*p* < 0.05) in *H* values in haddock and herring. Haddock and herring can be categorised as Ino-forming species as they both showed lower *H* values compared to *K* values and the ratios of Ino to Hx are more than 5:1 (Ehira & Uchiyama, 1973). The rate of change in *H* values also suggests that pressure treatment might delay the degradation of Ino to Hx and thus slow down the rate of accumulation of Hx, and therefore might slow down the rate of fish spoilage.

The greatest shelf-life extension in haddock, with an estimated shelf-life based on *H* values of 74.4 days of haddock treated at 300 MPa for 3 min. Herring treated at 300 MPa for 1 min had a prolonged shelf life up to 49.1 days based on *H* values. In contrast, control samples of haddock and herring were considered to have reached their *H* values shelf-life by 9.4 and 9.1 days respectively. In view of the extensive time required in the pressure treated haddock and herring to reach an *H* values of 60%, it seems most unlikely that such a high *H* value is inappropriate for estimating the shelf-life of pressure treated fish. With respect to the microbiological shelf life of high pressure treated haddock and herring (Karim et al., 2011) a more appropriate *H* values limit for such haddock and herring would be 30%. This would correspond to a shelf-life of ~5 days for the controls of both species. The shelf-life of high pressure treated haddock would be extended to between 6 and 14 days, and herring from 8 to 12 days when subjected to 200 and 250 MPa for 1 or 3 min. However, shelf-life for herring treated at 300 MPa for 1 or 3 min might be longer.

*F* values were calculated to estimate the initial deterioration of fish. Both pressure level and hold time had no significant effect on *F* values in either species. *F* values decreased significantly (*p* < 0.05) with prolonged storage. Gill et al. (1987) suggested that *F* values are most appropriate for fish freshness assessment especially in Ino-forming species. From the estimated curve that limited *F* values to 10%, haddock were predicted to have started to deteriorate by day 5 in controls and between day 5 and 7 in pressure-treated samples. In addition, control herring were estimated to have deteriorated noticeably by day 6 and between days 6 and 10 in pressure treated samples. These *F* value estimates of shelf-life were in many ways similar to those estimates given by *K* values. Since *F* values rely on the determination of the ratio of IMP to sum of IMP, Ino and Hx, this provides a simpler measure of freshness than the more complex *K* value determination and is recommended as a useful alternative measure of the freshness of fish.

High pressure processing decreased the soluble protein content of the crude enzyme extracts of both fish species (*p* < 0.05). Specifically, soluble protein concentration was also significantly decreased (*p* < 0.05) with increasing pressure level; at 200 MPa and above in haddock, and at 400 MPa and above in herring. Michel & Autio (2001) reported that HPP may lead to changes in the solubility of protein which was due to conformational changes affecting the functionality of the protein. Such alterations influence the quality and organoleptic attributes of foods (Kinsella, 1981).

Moderate pressures (100–300 MPa) are often effective for dissociating protein oligomers and aggregates, whereas relatively higher pressures (>300 MPa) are typically required for the denaturation of proteins (Hawley, 1971; Randoplh, Seefeldt & Carpenter, 2002; Silva & Weber, 1993; Silva, Foguel & Royer, 2001; Zipp & Kauzmann, 1973). HP affects the properties related to protein-protein interactions by the disruption of non-covalent interactions within the protein molecules and the subsequent reformation of intramolecular and intermolecular bonds within or between these protein molecules (Messens, Van Camp & Huyghebaert, 1997). The native structure of globular protein is stabilised by non-covalent (ionic, hydrogen and hydrophobic) and covalent (disulphide) bonds. Messens, Van Camp & Huyghebaert (1997) stated that electrostatic interactions (ionic bonds) that occur between ionised groups of amino acid side chains and from the C- and N-terminal amino acids in the polypeptide backbones of proteins can be disrupted by high pressure.

Hydrophobic interactions that maintain and stabilise the quaternary structure of proteins are considered to be the most sensitive to pressure (Lullien-Pellerin & Balny, 2002). A moderate pressure of <150 MPa, oligomeric proteins can be dissociated and accompanied by a large decrease in volume equivalent to 500 ml mol^−1^. These dissociations were followed by subunit aggregation and precipitation. Silva & Weber (1993) reported that pressures of around 150–200 MPa may lead to unfolding of protein monomers and re-association of subunits from dissociated oligomers. It has also been suggested that pressure-dissociated protein subunits undergo time-dependent conformational changes (Lullien-Pellerin & Balny, 2002). Dissociation of the tertiary structure of proteins has been found after pressure treatments beyond 200 MPa. In addition, the volume and compressibility changes during denaturation also occur after pressure treatments at 400–800 MPa (Lullien-Pellerin & Balny, 2002). This is followed by the reversible unfolding of proteins. Massons (1992) added that protein denaturation is a complex process involving intermediate forms leading to multiple denatured forms. It is thought that secondary structure dissociation occurs at 300–700 MPa and approaches non-reversible denaturation depending on the rate of compression and on the extent of secondary structure rearrangements (Balny & Masson, 1993).

In the current study, the protein content of the crude enzyme extracts of haddock decreased by 14.6% and 59.4% of the control values at 100 and 600 MPa,. Likewise, 7.1% and 67.7% of protein solubility was lost in the crude enzyme extracts of herring under the same conditions. 5′-NT activity in both haddock and herring was significantly decreased (*p* < 0.05) by high pressure treatment. In haddock, only 3.6% of 5′-NT activity was lost at 100 MPa but this increased markedly to 38–45% after pressure treatment between 200 and 600 MPa. However, 5′-NT activity in herring appeared to be more resistant to the effects of high pressure, only showing an obvious loss of 13.2% after treatment at 400 MPa and an eventual loss of 47.4% at 600 MPa. NP activity in both haddock and herring, although much lower than that of 5′-NT, also decreased significantly (*p* < 0.05) following high pressure treatment. The NP activity in haddock decreased by 40–60% after treatment at 200–600 MPa. In herring, NP activity decreased by 35.3–70.6% after pressure treatment at 100–600 MPa.

It is clear that a higher 5′-NT activity in both fish species compared to NP activity will promote a greater rate of conversion of IMP to Ino, whereas the accumulation of Hx through soluble NP activity would be relatively slow by comparison. Hx accumulated steadily throughout storage in both species of fish irrespective of pressure treatment. However, in this study, NP activity was determined within 1–2 days after the fish were harvested. At this stage, fish are considered fresh and the bacteria invasions considered to be relatively low. Thus, it is most likely that the conversion of Ino to Hx during chilled storage of haddock and herring is predominantly due to the activity of bacterial NP enzymes. Therefore, while autolytic conversion of IMP to Ino by endogenous 5′-NT predominates in the earliest stages of storage, both bacterial and endogenous NP enzymes are probably responsible for the gradual accumulation of Hx in fish, with spoilage bacteria greatly enhancing the production of Hx from Ino during chilled storage.

## Conclusion

High pressure processing was shown to delay nucleotide degradation and inhibit the accumulation of Hx that leads to the formation of uric acid in stored fish. In estimating both haddock and herring shelf-life based on nucleotide degradation, three approaches were compared in this study; *K* values, *H* values and *F* values. Recommended shelf-lives of haddock and herring based on *K* values and *F* values gave similar results and are considered superior to the use of recommended *H* values which grossly overestimated the shelf-life of pressure treated fish based on measurements of Hx, which is produced at a much lower rate than normal in pressure treated fish. Using *K* values data, high pressure treatment at 200 MPa for 3 min gave the longest shelf-life extension to haddock and herring compared to other pressure treatments. The shelf-lives of haddock and herring pressure treated at 200 MPa for 3 min were prolonged to 7.1 and 10.7 days respectively. HPP was effective in lowering the activity of 5-nucleotidase and nucleoside-phosphorylase in haddock and herring. The extent of inactivation was directly related to pressure level and is most likely due to changes in the structural conformation of these enzymes. This inactivation might also reduce the production of inosine and hypoxanthine which are associated with the progressive loss of desirable fresh fish flavour.

## Supplemental Information

10.7717/peerj.7527/supp-1 Supplemental Information 1 Nucleotide degradation of haddock and herring in high pressure processing during 14 days of storage.

10.7717/peerj.7527/supp-2 Supplemental Information 2 Nucleotide degradation of herring during 14 days of chilled storage.

10.7717/peerj.7527/supp-3 Supplemental Information 3 Nucleotide degradation of haddock during 14 days of chilled storage.

## Additional Information and Declarations

### Data Availability

The following information was supplied regarding data availability: The raw data are available as Supplemental Files.
