# Supplementary material for: Determination of nucleotide and enzyme degradation in haddock (Melanogrammus aeglefinus) and herring (Clupea harengus) after high pressure processing
Source: PeerJ. 2019 Aug 27;7:e7527. doi: 10.7717/peerj.7527 (PMC6716499; doi:10.7717/peerj.7527)
Supplement: Supplemental Information 2 [file peerj-07-7527-s002.docx]

|  |  | ATP umole/g | ADP umole/g | AMP umole/g | IMP umole/g | INO umole/g | HX umole/g |
| --- | --- | --- | --- | --- | --- | --- | --- |
| 0 Day | Control | 0.09 | 0.10 | 0.20 | 5.66 | 1.13 | 0.51 |
|  | 200 MPa 1 min | 0.09 | 0.07 | 0.23 | 5.88 | 1.18 | 0.75 |
|  | 200 MPa 3 min | 0.08 | 0.07 | 0.17 | 5.48 | 1.10 | 0.53 |
|  | 250 MPa 1 min | 0.07 | 0.09 | 0.15 | 5.15 | 1.03 | 0.73 |
|  | 250 MPa 3 min | 0.10 | 0.06 | 0.19 | 5.64 | 1.13 | 0.55 |
|  | 300 MPa 1 min | 0.06 | 0.06 | 0.18 | 6.05 | 1.21 | 0.42 |
|  | 300 MPa 3 min | 0.07 | 0.04 | 0.10 | 6.50 | 1.30 | 0.58 |
| 2 Day |  |  |  |  |  |  |  |
|  | Control | 0.08 | 0.11 | 0.39 | 2.36 | 0.47 | 2.26 |
|  | 200 MPa 1 min | 0.06 | 0.13 | 0.29 | 4.46 | 0.89 | 1.63 |
|  | 200 MPa 3 min | 0.10 | 0.13 | 0.24 | 4.05 | 0.81 | 1.25 |
|  | 250 MPa 1 min | 0.05 | 0.09 | 0.28 | 5.27 | 1.05 | 1.51 |
|  | 250 MPa 3 min | 0.09 | 0.15 | 0.32 | 4.62 | 0.92 | 1.51 |
|  | 300 MPa 1 min | 0.06 | 0.09 | 0.40 | 2.91 | 0.58 | 2.04 |
|  | 300 MPa 3 min | 0.05 | 0.03 | 0.18 | 4.92 | 0.98 | 1.58 |
| 4 Day |  |  |  |  |  |  |  |
|  | Control | 0.16 | 0.23 | 0.23 | 4.82 | 0.96 | 2.11 |
|  | 200 MPa 1 min | 0.15 | 0.22 | 0.21 | 4.60 | 0.92 | 1.92 |
|  | 200 MPa 3 min | 0.11 | 0.20 | 0.16 | 3.56 | 0.71 | 1.84 |
|  | 250 MPa 1 min | 0.12 | 0.14 | 0.09 | 4.91 | 0.98 | 1.73 |
|  | 250 MPa 3 min | 0.08 | 0.21 | 0.19 | 5.41 | 1.08 | 1.93 |
|  | 300 MPa 1 min | 0.09 | 0.19 | 0.09 | 4.94 | 0.99 | 2.17 |
|  | 300 MPa 3 min | 0.09 | 0.06 | 0.06 | 3.75 | 0.75 | 1.81 |
| 6 Day |  |  |  |  |  |  |  |
|  | Control | 0.08 | 0.17 | 0.18 | 1.48 | 0.30 | 5.72 |
|  | 200 MPa 1 min | 0.11 | 0.17 | 0.11 | 3.85 | 0.77 | 2.78 |
|  | 200 MPa 3 min | 0.09 | 0.14 | 0.05 | 3.20 | 0.64 | 2.31 |
|  | 250 MPa 1 min | 0.10 | 0.11 | 0.04 | 3.28 | 0.66 | 1.84 |
|  | 250 MPa 3 min | 0.10 | 0.19 | 0.02 | 6.82 | 1.36 | 1.87 |
|  | 300 MPa 1 min | 0.11 | 0.15 | 0.09 | 1.61 | 0.32 | 1.83 |
|  | 300 MPa 3 min | 0.05 | 0.04 | 0.02 | 4.08 | 0.82 | 1.86 |
| 10 Day |  |  |  |  |  |  |  |
|  | Control | 0.10 | 0.17 | 0.55 | 1.13 | 0.23 | 8.27 |
|  | 200 MPa 1 min | 0.10 | 0.16 | 0.64 | 0.75 | 0.15 | 6.69 |
|  | 200 MPa 3 min | 0.10 | 0.18 | 0.55 | 1.39 | 0.28 | 3.65 |
|  | 250 MPa 1 min | 0.09 | 0.14 | 0.36 | 1.31 | 0.26 | 2.55 |
|  | 250 MPa 3 min | 0.11 | 0.19 | 1.23 | 1.12 | 0.22 | 5.60 |
|  | 300 MPa 1 min | 0.12 | 0.17 | 0.45 | 1.25 | 0.25 | 2.60 |
|  | 300 MPa 3 min | 0.15 | 0.04 | 0.25 | 1.59 | 0.32 | 2.39 |
| 14 Day |  |  |  |  |  |  |  |
|  | Control | 0.16 | 0.16 | 0.56 | 0.23 | 0.05 | 10.43 |
|  | 200 MPa 1 min | 0.05 | 0.19 | 0.39 | 0.84 | 0.17 | 8.65 |
|  | 200 MPa 3 min | 0.06 | 0.18 | 0.42 | 0.31 | 0.06 | 6.51 |
|  | 250 MPa 1 min | 0.05 | 0.11 | 0.34 | 1.51 | 0.30 | 2.33 |
|  | 250 MPa 3 min | 0.05 | 0.19 | 0.48 | 0.85 | 0.17 | 5.07 |
|  | 300 MPa 1 min | 0.08 | 0.14 | 0.37 | 0.82 | 0.16 | 4.38 |
|  | 300 MPa 3 min | 0.04 | 0.04 | 0.28 | 0.74 | 0.15 | 2.53 |

Herring Nucleotides degradation
