# Supplementary material for: Determination of nucleotide and enzyme degradation in haddock (Melanogrammus aeglefinus) and herring (Clupea harengus) after high pressure processing
Source: PeerJ. 2019 Aug 27;7:e7527. doi: 10.7717/peerj.7527 (PMC6716499; doi:10.7717/peerj.7527)
Supplement: Supplemental Information 3 [file peerj-07-7527-s003.docx]

|  |  | ATP | ADP | AMP | IMP | INO | HX |
| --- | --- | --- | --- | --- | --- | --- | --- |
| 0 Day | Control | 0.06 | 0.43 | 0.29 | 5.69 | 3.66 | 0.67 |
|  | 200 MPa 1 min | 0.20 | 0.45 | 0.25 | 6.96 | 4.32 | 0.77 |
|  | 200 MPa 3 min | 0.15 | 0.36 | 0.24 | 6.45 | 3.85 | 0.43 |
|  | 250 MPa 1 min | 0.07 | 0.16 | 0.08 | 6.88 | 4.06 | 0.74 |
|  | 250 MPa 3 min | 0.09 | 0.43 | 0.24 | 6.32 | 3.97 | 0.55 |
|  | 300 MPa 1 min | 0.10 | 0.28 | 0.22 | 4.06 | 5.49 | 0.74 |
|  | 300 MPa 3 min | 0.03 | 0.06 | 0.27 | 5.01 | 2.96 | 1.05 |
| 2 Day |  |  |  |  |  |  |  |
|  | Control | 0.12 | 0.46 | 0.32 | 5.30 | 4.16 | 0.86 |
|  | 200 MPa 1 min | 0.12 | 0.38 | 0.34 | 3.15 | 5.77 | 1.14 |
|  | 200 MPa 3 min | 0.13 | 0.36 | 0.27 | 3.23 | 6.57 | 0.55 |
|  | 250 MPa 1 min | 0.04 | 0.12 | 0.10 | 4.70 | 4.85 | 1.08 |
|  | 250 MPa 3 min | 0.09 | 0.34 | 0.33 | 2.63 | 5.31 | 1.17 |
|  | 300 MPa 1 min | 0.07 | 0.23 | 0.22 | 3.98 | 6.01 | 0.70 |
|  | 300 MPa 3 min | 0.04 | 0.05 | 0.19 | 3.62 | 6.20 | 0.83 |
| 4 Day |  |  |  |  |  |  |  |
|  | Control | 0.06 | 0.41 | 0.41 | 2.44 | 5.42 | 1.98 |
|  | 200 MPa 1 min | 0.15 | 0.44 | 0.32 | 2.30 | 6.09 | 1.34 |
|  | 200 MPa 3 min | 0.08 | 0.36 | 0.28 | 1.88 | 6.39 | 1.29 |
|  | 250 MPa 1 min | 0.07 | 0.18 | 0.14 | 1.26 | 7.16 | 1.61 |
|  | 250 MPa 3 min | 0.08 | 0.41 | 0.29 | 3.56 | 5.01 | 1.14 |
|  | 300 MPa 1 min | 0.09 | 0.23 | 0.24 | 2.70 | 6.68 | 1.84 |
|  | 300 MPa 3 min | 0.04 | 0.05 | 0.20 | 1.81 | 7.29 | 2.05 |
| 6 Day |  |  |  |  |  |  |  |
|  | Control | 0.18 | 0.46 | 0.40 | 1.22 | 4.75 | 3.34 |
|  | 200 MPa 1 min | 0.11 | 0.38 | 0.31 | 0.59 | 3.92 | 1.75 |
|  | 200 MPa 3 min | 0.07 | 0.15 | 1.17 | 2.78 | 4.01 | 2.18 |
|  | 250 MPa 1 min | 0.03 | 0.10 | 0.10 | 3.34 | 7.25 | 4.92 |
|  | 250 MPa 3 min | 0.16 | 0.96 | 0.71 | 1.74 | 5.02 | 2.29 |
|  | 300 MPa 1 min | 0.95 | 0.41 | 0.15 | 2.38 | 6.56 | 2.28 |
|  | 300 MPa 3 min | 0.02 | 0.32 | 0.17 | 1.98 | 6.45 | 2.11 |
| 10 Day |  |  |  |  |  |  |  |
|  | Control | 0.10 | 0.49 | 0.39 | 0.25 | 3.17 | 8.54 |
|  | 200 MPa 1 min | 0.11 | 0.49 | 0.42 | 0.46 | 6.62 | 3.55 |
|  | 200 MPa 3 min | 0.07 | 0.40 | 0.28 | 0.68 | 6.92 | 3.04 |
|  | 250 MPa 1 min | 0.04 | 0.11 | 0.13 | 1.10 | 6.97 | 2.36 |
|  | 250 MPa 3 min | 0.10 | 0.46 | 0.36 | 0.60 | 6.99 | 3.13 |
|  | 300 MPa 1 min | 0.07 | 0.26 | 0.18 | 0.47 | 7.45 | 2.61 |
|  | 300 MPa 3 min | 0.05 | 0.02 | 0.07 | 0.19 | 5.87 | 2.61 |
| 14 Day |  |  |  |  |  |  |  |
|  | Control | 0.24 | 0.50 | 0.37 | 0.06 | 1.32 | 9.93 |
|  | 200 MPa 1 min | 0.17 | 0.49 | 0.39 | 0.10 | 4.91 | 5.44 |
|  | 200 MPa 3 min | 0.11 | 0.49 | 0.28 | 0.34 | 5.89 | 3.96 |
|  | 250 MPa 1 min | 0.05 | 0.12 | 0.09 | 1.07 | 6.11 | 2.72 |
|  | 250 MPa 3 min | 0.16 | 0.44 | 0.35 | 0.18 | 4.11 | 5.05 |
|  | 300 MPa 1 min | 0.10 | 0.35 | 0.27 | 0.09 | 6.97 | 3.06 |
|  | 300 MPa 3 min | 0.02 | 0.04 | 0.06 | 0.11 | 7.58 | 3.50 |

Haddock Nucleotides degradation
